# Attitudes toward digital pill systems for antiretroviral therapy adherence among pregnant and postpartum women living with HIV in South Africa: a qualitative study

**DOI:** 10.3389/fpsyt.2025.1667547

**Published:** 2025-12-04

**Authors:** Madison R. Fertig, Jasper S. Lee, Stephan Rabie, Yanga Tshukuse, Mfanelo Ncukana, Devisi A. Ashar, Chris Carnes, Conall O’Cleirigh, John A. Joska, Peter R. Chai, Amelia M. Stanton

**Affiliations:** 1 Department of Psychological and Brain Sciences, Boston University, Boston, MA, United States; 2 Department of Psychiatry, Massachusetts General Hospital, Boston, MA, United States; 3 Department of Psychiatry, Harvard Medical School, Boston, MA, United States; 4 Department of Emergency Medicine, Brigham and Women’s Hospital, Boston, MA, United States; 5 HIV Mental Health Research Unit, Division of Neuropsychiatry, Faculty of Health Sciences, University of Cape Town, Cape Town, South Africa; 6 etectRx, Gainesville, FL, United States; 7 Fenway Institute, Boston, MA, United States; 8 Koch Institute for Integrative Cancer Research, Massachusetts Institute of Technology, Cambridge, MA, United States

**Keywords:** digital pill technology, HIV, ART, pregnancy, postpartum, South Africa

## Abstract

**Introduction:**

In South Africa (SA), 22.6% of cisgender women aged 15-49 are living with HIV; of these, one in three are pregnant. Perinatal adherence to antiretroviral therapy (ART) is critical to prevent mother to child transmission (MTCT) of HIV. However, there are numerous barriers to adherence (e.g., pregnancy/postpartum physical symptoms, poor mental health, low social support, structural barriers). In the US, digital pill systems (DPS; i.e., ingestible radiofrequency sensors integrated into gelatin capsules that over-encapsulate medications) have been used to measure adherence to preexposure prophylaxis (PrEP) among men who have sex with men. Interventions that incorporate DPS may facilitate ART adherence and reduce risk of MTCT in SA, but preliminary acceptability has not yet been explored in this context.

**Methods:**

Thirty pregnant (n=15) and postpartum (n=15) women living with HIV who reported ART adherence challenges were introduced to DPS and completed qualitative interviews. Qualitative data were analyzed via rapid qualitative analysis.

**Results:**

Participants reported that they missed approximately 3.0 (SD=2.1) ART doses in the past 30 days. Most participants found the overall DPS concept and its components to be acceptable, including ingesting a radiofrequency sensor and wearing a digital pill Reader, which collects adherence data from the digital pill and relays it to a smartphone via a linked app, as a lanyard. They suggested that DPS would improve their adherence and increase accountability to prevent HIV transmission to their infants. Participants who disclosed their HIV status to close friends and family viewed wearing the Reader for several minutes a day to be acceptable and appreciated that providers could access adherence data. They also expressed that the smartphone app would provide helpful reminders to collect and take their ART. However, for both pregnant and postpartum participants, the primary barrier to likely use of the DPS for themselves or others was the risk of involuntary HIV status disclosure by wearing the visible Reader.

**Discussion:**

Future research should explore ideal Reader systems to facilitate use among this population in SA, especially among persons who report low ART adherence or have unsuppressed HIV RNA when presenting for antenatal care.

## Introduction

Cisgender women, specifically those who are pregnant or postpartum, face a disproportionate burden of HIV acquisition in southern Africa, such that 63% of new infections occur among cisgender girls and women (1). In South Africa (SA), about one third of pregnant women who attend public antenatal clinics are living with HIV (1). Perinatal adherence to antiretroviral therapy (ART) is critical to prevent mother to child transmission (MTCT) of HIV, but rates of ART adherence during the peripartum period remain suboptimal. Adherence rates are typically higher during pregnancy than after women give birth—often due to lower prioritization of postpartum health compared to prenatal health, increased financial stress, and immediate newborn demands—despite continued high risk of transmission during breastfeeding (2). On average, 73.5% of pregnant women are adherent to ART, while adherence levels are lower among postpartum women (~53%) (3), highlighting suboptimal viral load suppression among this population and contributing to increased risk of MTCT (4). This disparity suggests that developing adherence measurement and support systems for pregnant and postpartum women with suboptimal ART adherence in SA may be an important strategy to end the HIV epidemic.

There are multi-level barriers during pregnancy and postpartum that contribute to poor ART adherence. At the individual level, common pregnancy symptoms, ART side effects, and mental health symptoms can interfere with ART adherence (5–8). For example, about 70-85% of pregnant women experience nausea and vomiting induced by pregnancy, symptoms that can be exacerbated by ART, thereby contributing to nonadherence (3, 4). Common mental health disorders, particularly depression, has been shown to affect up to 47% of peripartum women in SA (9), likely driven by poverty, unplanned pregnancy, and intimate partner violence (IPV) (10, 11). Depression may lead to social isolation, hopelessness, and impaired problem-solving strategies (8), all of which can weaken social support and lead to reduced ART use (12, 13). Experiencing IPV may also act as a barrier to ART adherence. In southern Africa, rates of IPV among pregnant women living with HIV ranges from 18-63%. These experiences can increase vulnerability to posttraumatic stress symptoms (14) and contribute to non-disclosure of HIV status, missing clinic appointments, and lower ART adherence (15). Structural barriers, such as clinic non-attendance and HIV stigma among providers working in the clinic, can also contribute to reduced ART adherence among pregnant and postpartum women. In low- and middle-income countries (LMICs), individuals may need to prioritize working over clinic attendance, especially if jobs are scarce, which negatively impacts ART adherence (16, 17). Additionally, stigmatizing practices within healthcare settings, like separate waiting areas for people living with HIV (PWH) or the use of distinctively colored medical chart folders for PWH, may unintentionally disclose HIV status publicly (8, 18). The risk of public disclosure creates a barrier to healthcare engagement and may also reduce ART adherence.

To better measure ART adherence and provide adherence support, many strategies have been used. These include indirect methods like self-report, pill counts, and pharmacy refill data. These strategies may be inaccurate in determining actual pill-taking, which may be particularly problematic during pregnancy and postpartum among patients who have increased risk of MTCT. Providers usually depend on patients’ self-reported ART adherence, which is often subject to recall bias (19). Viral load testing is also critical, but delays from specimen collection to clinical response and treatment prevents a prompt response to poor adherence in many LMICs. Technology assisted monitoring with electronic pill storage, smart pill bottles that infer adherence with opening of the pill bottle, and smart packaging to indicate use of blister packs facilitate assessment of the time and date at which the device is opened have been implemented to support ART adherence (20). While these proxy markers of adherence have shown some acceptability, these technologies do not track direct pill swallowing. As such, few direct measures of ART adherence, outside of directly observed or video observed therapy, have been employed in LMICs.

**Figure 1:**
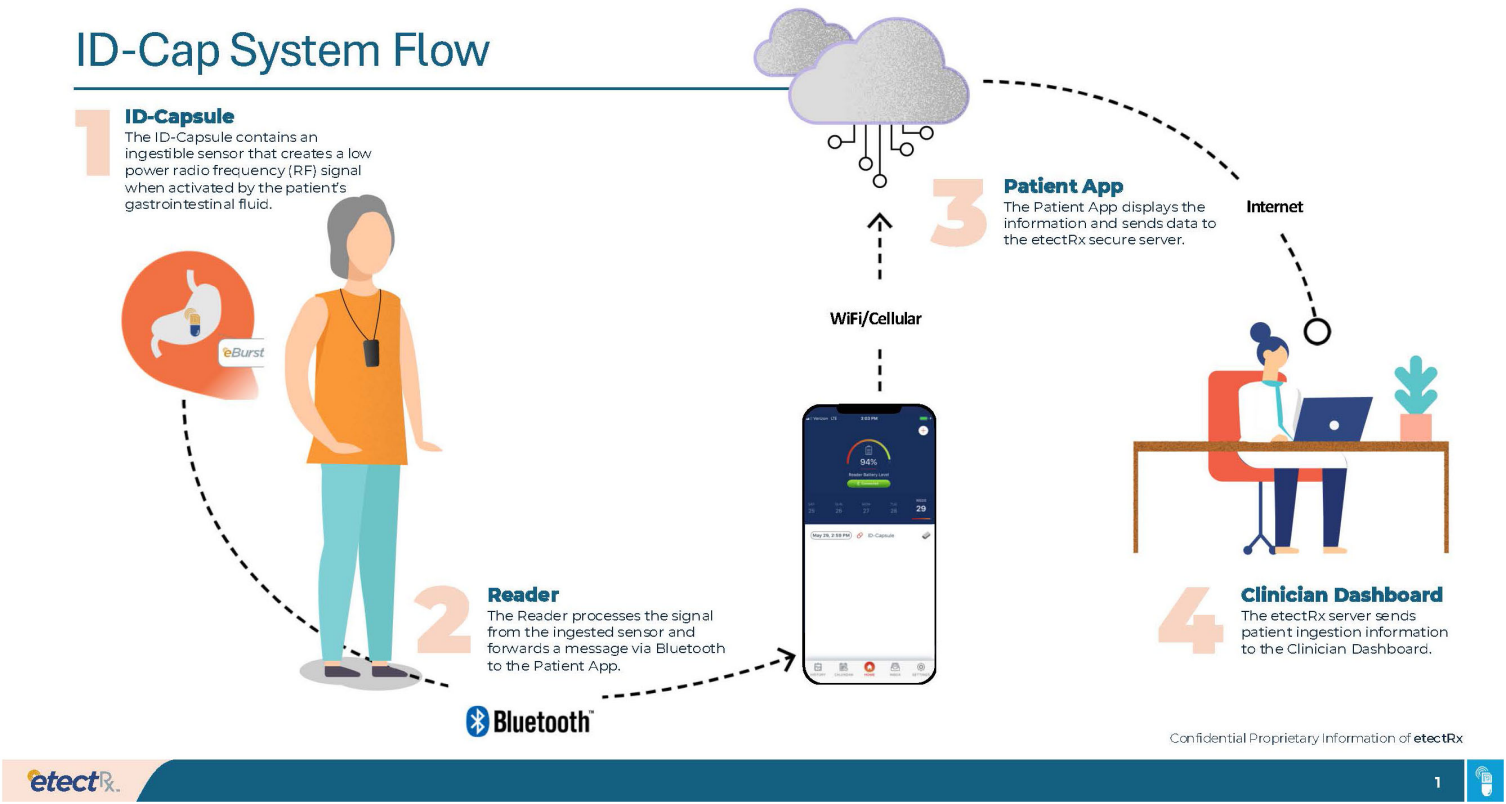
ID-Cap System Flow diagram illustrating the process of the digital pill system from ingestion to clinician notification.

In the United States, digital pill systems (DPS) have been tested over 16 years as an objective metric of medication adherence and have been successfully utilized to measure preexposure prophylaxis (PrEP) and ART adherence in research settings. DPS are ingestible radiofrequency (RF) sensors integrated into gelatin capsules that over-encapsulate medications (21); when swallowed, stomach acid activates the RF sensor in the digital pill, sending a signal to the wearable Reader (i.e., a digital pill necklace receiver that collects the radio signal from an activated digital pill) that the pill has been successfully taken (**Figure 1**). The Reader then connects to a linked phone app via Bluetooth and provides adherence data for both the patient and healthcare team. In a sample of men who have sex with men (MSM) with substance use disorders in the US, DPS was deemed acceptable and feasible for measuring real-time PrEP adherence. Participants were able to effectively integrate DPS into their daily lives and routines (22). When used among PWH in the US, DPS was associated with lower HIV RNA viral load (23). This strategy could be translated into the SA context as a novel, direct method to better assess and respond to suboptimal ART adherence in real time, especially in the peripartum period during which optimal adherence may prevent MTCT. The DPS (ID-Cap System; etectRx) has been approved by the Food and Drug Administration (FDA) for use in humans, and has been tested in several US-based studies (24–26), but has not been used in pregnant or postpartum women.

Attitudes toward DPS have not yet been explored in SA. Doing so among pregnant and postpartum women with self-reported reduced ART adherence will provide critical information on their willingness to use DPS to reduce HIV transmission risk to their infants. Therefore, the purpose of the study was to explore the acceptability and feasibility of the DPS technology to monitor ART adherence among pregnant and postpartum women living with HIV in SA.

## Methods

### Study procedures

Pregnant and postpartum individuals living with HIV were recruited from two antenatal clinics embedded in larger community clinics, in Khayelitsha, a peri-urban settlement outside of Cape Town, SA, where study staff had existing relationships with clinic personnel. The study explored the acceptability of DPS for measuring and providing feedback on ART adherence during these periods. Research assistants who recruited participants and conducted interviews were trained by US- and SA-based project staff in a two-day in-person training, which included learning the study materials (i.e., study protocol, informed consent form, questionnaire, and interview guide) and role-playing study visits with principal investigators, supervisors, and each other. Potential participants were approached by study research assistants while waiting in queue at the clinic. Clinic staff were also informed of the study inclusion criteria to refer eligible patients. Clinic patients who expressed interest were invited to be screened for eligibility by a research assistant in a private space at the clinic. Eligibility criteria for pregnant participants were as follows: (1) pregnant (at any gestational age) and living with HIV (confirmed via medical chart review); (2) aged 18 years or older; (3) receiving treatment for HIV; (4) self-reported non-adherence to ART (any missed doses in the past 30 days); and (5) able to complete study procedures in isiXhosa or English. Eligibility criteria for postpartum participants were the same, except these participants had given birth within the past six months and were currently breastfeeding. Those who were deemed to be eligible were given the option of completing the informed consent process and continuing to study participation that day or returning on a later date. Informed consent was obtained in the same private setting, where the research assistant reviewed the consent form with the potential participant, obtained written consent from those who agreed to participate, and provided participants with a copy for their records. This study was approved by the ethics committees at Massachusetts General Hospital (ethics reference #2023P000088) and University of Cape Town (UCT) (HREC REF: 007/2023). A collaboration agreement was also established between UCT and Boston University.

A total of 48 pregnant and postpartum women were screened for eligibility, 21 pregnant and 20 postpartum individuals met inclusion criteria. Of the 41 individuals who were eligible, 30 completed a brief questionnaire and qualitative interview in a private space at the clinic where they were recruited. The questionnaire assessed basic demographic information, pregnancy characteristics, self-reported ART adherence, and willingness to use DPS [adapted from System Usability Scale (27)].

A semi-structured interview guide was developed in accordance with guidelines specified by Miles and Huberman (28) to explore (1) attitudes toward digital pill technology; (2) feasibility and acceptability of using DPS during pregnancy and the postpartum period; (3) attitudes toward DPS privacy practices and data sharing; and (4) future recommendations for DPS. During the interview, participants also watched an instructional video on the DPS that clarified the ways in which the system monitors ART adherence and ensures the safety of the user. The digital pills, gelatin capsules, and readers were also presented in-person so participants could interact with the DPS. Participants were encouraged to take apart the gelatin capsule and inspect the radiofrequency emitter.

### Analysis

Participant interviews were audio-recorded, transcribed, and translated from isiXhosa to English by SA-based research assistants (YT, MN). Qualitative data were analyzed using a rapid qualitative analysis (RQA) approach. Before data collection, the second author (JSL) developed a matrix framework to organize primary domains and subdomains aligned with the interview guide. Transcripts were reviewed, and relevant excerpts were entered into the corresponding matrix cells in Microsoft Excel. JSL initiated the coding process with seven transcripts, and the first author (MRF) continued reviewing transcripts, refining the framework by adding subdomains to capture content more comprehensively. Throughout the process, the team held biweekly meetings to discuss emerging content themes and ensure consistent and accurate interpretation of the data between SA–based research assistants and data analysts. All co-authors reviewed, discussed, and agreed upon finalized themes and sub-themes prior to manuscript submission. The final synthesis focused on identifying patterns and themes across domains and linking findings to broader research priorities and existing literature.

## Results

The sample consisted of 30 pregnant (n=15) and postpartum (n=15) women who had an average age of 31 years (SD = 6.2). Pregnant participants had an average gestational age of 23.3 weeks (SD = 11.3), while postpartum participants averaged 11.3 weeks post-delivery (SD = 9.1). Participants on average missed three doses of ART in the past 30 days (SD = 2.1) and 28 participants (93.3%) reported that they did not hide their HIV medication from others. Full demographics details are displayed in **Table 1**. Most participants indicated that the DPS would be very useful and expressed high levels of willingness to use the technology to support their ART adherence. Participants’ full quantitative responses to their willingness to use DPS are displayed in **Table 2**.

**Table 1:** Participant demographics.

Demographics	M, SD
Age (M, SD)	31.0 (6.2)
Gestational age (M, SD)	23.3 (11.7)
Weeks, postpartum (M, SD)	11.3 (9.1)
Gestational age when presenting for ANC (M, SD)	10.0 (5.5)
Number of lifetime pregnancies (M, SD)	2.5 (1.3)
Number of live births (M, SD)	1.9 (1.2)
Number of misses doses in the past 30 days (M, SD)	3.0 (2.1)
Education	(n, %)
Grade 9/Std 7	4 (13.3)
Grade 10/Std 8	2 (6.7)
Grade 11/Std 9	13 (43.3)
Grade 12/Std 10	9 (30)
Vocational Training	2 (6.7)
Hide HIV medication	(n, %)
No	28 (93.3)

**Table 2:** Participants’ willingness to use DPS.

Item	Strongly disagree	Disagree	Neither agree nor disagree	Agree	Strongly agree
I think that I would use this system frequently.	0	0	0	25 (83.3)	5 (16.7)
I think that I would find the system unnecessarily complex.	2 (6.7)	12 (40)	1 (3.3)	14 (46.7)	1 (3.3)
I think the system would be easy to use.	1 (3.3)	2 (6.7)	1 (3.3)	23 (76.7)	3 (10)
I think that I would need the support of a technical person to be able to use this system.	0	10 (33.3)	1 (3.3)	17 (56.7)	2 (6.7)
I think that the various functions in this system are well integrated.	1 (3.4)	0	1 (3.4)	25 (86.2)	2 (6.9)^1^
I think there is too much inconsistency in the system.	1 (3.3)	20 (66.7)	4 (13.3)	4 (13.3)	0
I would imagine that most people would learn to use this system very quickly.	1 (3.3)	1 (3.3)	0	23 (76.7)	5 (16.7)
I think the system would be very cumbersome to use.	1 (3.3)	26 (86.7)	1 (3.3)	2 (6.7)	0
I would feel very confident using the system if it were given to me.	1 (3.3)	0	0	25 (83.3)	4 (13.3)
I need to learn a lot of things before I can get going with the system.	0	7 (23.3)	0	19 (63.3)	4 (13.3)
	Not at all useful	Slightly Useful	Moderately useful	Very useful	Extremely useful
Helping you adhere to ART	0	0	0	26 (86.7)	4 (13.3)
Helping someone who is brand new to taking ART to adhere to ART	0	0	0	26 (86.7)	4 (13.3)
Helping to hold you accountable for your ART adherence	0	0	0	26 (86.7)	4 (13.3)
Helping to access information about your past ART adherence on your phone	0	0	0	26 (86.7)	4 (13.3)
Assisting the healthcare team in monitoring your ART adherence	0	0	0	25 (83.3)	5 (16.7)
	Not at all trusting	Slightly trusting	Moderately trusting	Very trusting	Extremely trusting
To what degree would you trust that the digital pill system would accurately provide information on your daily adherence to ART?	0	2 (6.9)	0	26 (86.7)	2 (6.9)
	Not at all willing	Slightly willing	Moderately willing	Very willing	Extremely willing
A wearable device paired with the digital pill (e.g., Fitbit or Apple Watch) that would collect biometric information (e.g., heart rate, respiratory rate, oxygen saturation, temperature) while you take your ART	0	0	2 (6.9)	25 (83.3)	3 (10)
Smartphone data (e.g., geographic location, battery level, text messaging, frequency of use of the app connected to the digital pill system)	0	0	1 (3.3)	26 (86.7)	3 (10)
Messages (e.g., Whatsapp) asking about things that get in the way of taking your ART	1 (3.3)	0	0	26 (86.7)	3 (10)
Phone calls asking about things that get in the way of taking your ART	1 (3.3)	1 (3.3)	0	25 (83.3)	3 (10)
Messages (e.g., Whatsapp) asking about your general daily activities and locations	0	0	0	27 (90)	3 (10)
Phone calls asking about your general daily activities and locations	1 (3.3)	0	0	26 (86.7)	3 (10)
Based on what you know about the digital pill, to what degree would you be willing to use digital pills to measure how you take your ART?	0	0	2 (6.9)	24 (80)	4 (13.3)

^1^One participant refused to answer

Our qualitative analysis revealed participants’ perceptions of the DPS, highlighting perceived benefits for ART adherence and challenges that may hinder use. Theme 1 suggests that DPS could improve adherence and increase accountability to prevent HIV transmission to their babies, emphasizing that (1) wearing the DPS reader for several minutes a day is acceptable and (2) sending adherence data to healthcare providers is also acceptable, especially to increase transparency about ART adherence and provide support. In general, participants agreed that a linked smartphone app would provide helpful reminders to collect and take ART (Theme 2). However, fear of disclosing HIV status by wearing the visible Reader was the primary barrier to likely use of the DPS (Theme 3).

### Theme 1: DPS would improve adherence and increase accountability to prevent HIV transmission to their infants

Both pregnant and postpartum participants viewed the DPS as a valuable tool not only for monitoring their own ART adherence, but also a way to keep their babies from acquiring HIV. One participant shared, *“That is another time that I think it is important, you know, to be adherent, because the baby depends on you, and you have to take your medication as well so that you don’t affect the baby. So, I think while breastfeeding, I would be highly motivated to take the digital pill or to use the technology.”* (postpartum, age 40). Similarly, a pregnant participant was thinking ahead to breastfeeding, noting that DPS would support her in keeping her baby safe, *“It would be a good thing, especially when you breastfeeding … the child will get something that will protect them that nothing may come in or harm the child.”* (pregnant, age 24).

Other participants expressed that being able to view adherence results would act as a motivator to continue daily ART use. One participant described the benefit of both feeling motivated to see her results and knowing she is keeping her baby safe. She said,

*“I feel like it would be helpful a lot because it tells you that you have taken your pill. So, what I love a lot about the digital, it tracks that you’ve taken it today and the week how did you go? So, it would help me a lot to take my pills on time even my baby to be like healthy … What would encourage me to take it because I want to see the results.”* (pregnant, age 28).

Additionally for pregnant and postpartum women who lack social support surrounding their ART adherence, DPS could provide instrumental support for consistent adherence. A postpartum participant expressed her optimism for DPS and that, despite not having adequate support, DPS could be capable of filling this gap during a high-risk period; she said,

*“I think the number of people who are defaulting is also going to drop, So it will be able to show if someone is taking their medication or not … I like that. It motivates you in it encourages you. And there is something that is going to remind you to take your medication, especially for people who do not have someone to motivate them or encourage them, or someone who lives alone and does not have someone to remind them to take their medication.”* (postpartum, age 33).

Participants generally found the DPS to be acceptable and motivating for improving adherence, with some suggesting that integrating it into clinical care could help reduce rates of treatment default.

#### Subtheme 1: Wearing the DPS reader for several minutes a day is acceptable among participants who have disclosed their HIV status to close friends and family

Pregnant and postpartum participants who have disclosed their HIV status to close friends and family viewed wearing the DPS Reader around their neck as acceptable. Many participants felt secure in knowing that wearing the Reader for a few minutes after taking their ART would keep them safe and adherent. One participant stated, *“No, I don’t mind using it, even if other people can see it, because I know why it is helping me.”* (postpartum, age 42). Participants discussed that HIV-related stigma in their close circles or in their broader community would not interfere with using the Reader. One participant who had disclosed her status to friends and family said,

*“Yes, I will use it in front of them. I’m hiding the normal stuff. If they want to make fun of it, I don’t really mind … Like I just said that now if they laugh at it, they will laugh, that is their own problem because I didn’t ask for it.”* (pregnant, age 29).

Some participants even mentioned that they would share what the Reader is used for because they are not afraid to disclose their status. One woman said,

*“I would wear the reader, there’s nothing wrong with it, and there’s nothing that would stop me from wearing it, since I’m not hiding anything from anyone in the house … No, I’m not the kind of person who is afraid to disclose their status, so I would wear it anyway, and when you ask me about it, I’ll tell you exactly what it is.”* (pregnant, age 28).

Participants who had acknowledged and shared their HIV status not only viewed the Reader as acceptable to wear, but were open to discussing it with others who inquired.

#### Subtheme 2: Sending adherence data to healthcare providers is acceptable to increase transparency about ART adherence and provide support

DPS would allow both patients and healthcare providers to access adherence data to improve adherence transparency with providers, as well as expedite necessary communication if adherence is poor. Most participants viewed provider access as beneficial for monitoring how well they are taking their ARTs. Participants also wanted their providers to see evidence of high adherence. One participant said, *“That would be a good thing, because they will see that I’m taking my medication correctly. I don’t skip.”* (pregnant, age 24). Another participant mentioned favoring provider access as a way for the provider to communicate if she did not take her ARTs, *“I like that the doctor can see how you can take your medication, and they would be able to remind you, if you did not take it”* (postpartum, age 35). Similarly, participants described provider involvement as a mechanism for offering assistance to improve their pill taking. One participant stated, *“There’s no problem with that at all (seeing adherence data). So that you guys can be able to tell me when I am not doing well maybe I don’t see it.”* (pregnant, age 36). In line with DPS improving motivation and accountability, participants viewed having their providers engage with their adherence data as its own motivator to continue DPS use. One said, “*I think it would make me also want to take my medication or adhere to your medication.”* (postpartum, age 18). Most participants were quite positive about the ways in which DPS would improve their overall ART adherence, specifying that concrete data to confirm protection of the baby and including providers in accessing adherence data increased their willingness to use the technology.

### Theme 2: The linked smartphone app would provide helpful reminders to collect and take ART

Most participants were intrigued by the smartphone app, primarily because they endorsed forgetting to take their ARTs at times or missed their clinic appointments. In that sense, the app would help prevent participants from questioning if they took their pills that day, leading to reduced stress levels. One participant said:

*“I think that would be helpful, because we are people who are forgetful, because sometimes you just wonder if you took a pill or did not take it. Because I myself sometimes wonder if I’ve taken my pill or I don’t I haven’t taken it. I asked myself, did I take it today. Did I not take it? So, I think that would be very helpful.”* (postpartum, age 40).

Similarly, another participant discussed that the app would make her feel happy that she was successfully adherent,

*“That would make me happy, that would make me happy, because I don’t get stressed when I know I’ve taken my medication, and when I know that I took my medication for whole month, And I did not forget I would be happy.”* (postpartum, age 42).

Participants were also interested in having the smartphone app link clinic appointments to the in-app calendar, including antenatal and HIV-related appointments, which would ensure that both types of appointments were attended and that patients would collect their ART on time. One participant shared, “*That will be very helpful so that I know that on a certain date I have an appointment and then I don’t forget*.*”* (pregnant, age 32). Another participant emphasized that receiving reminders and notifications about clinic appointments would reduce the chance that she will miss her appointments, “*Yes, that would be great, because I will not forget I would know that on this day I’m supposed to go get my medication.”* (postpartum, age 33). Ideally, the app would not only send reminders when a pill needs to be taken, but also when a dose has been missed. A participant shared this sentiment, *“I would like to receive a notification after I have taken my pill, and also a notification when I have forgotten to take my pill.”* (postpartum, age 31). Although many participants reported sharing a smartphone with a partner or family member, they generally did not mind receiving app notifications—even if their partner or family members saw. Overall, most participants were highly interested in the linked smartphone app because it offered a feasible way for them to stay notified, adherent, and less worried about missing doses or appointments.

### Theme 3: Fear of disclosing their HIV status by wearing the visible Reader was the primary barrier to likely use of the DPS

Although participants who had disclosed their status were not worried about wearing the Reader, there were a few who had not disclosed and were hesitant about wearing an object that clearly identified their HIV status to others. One participant shared, *“The only difficult part is that I think it would be hard for me to hide it, you know, at home, because that reader is a little bit big.”* (pregnant, age 25). Several participants also identified the Reader as the biggest barrier to DPS implementation because they would have to explain the device to others, which could expose them to enacted stigma. One woman said,

*“I think people who do not want to disclose their status and who are generally hiding their status would have a hard time with the culture using the reader because they would have to explain to their partners and their family about the things that they are doing, like wearing the reader and people would ask questions about what they are saying.”* (pregnant, age 40).

Wearing the Reader could also lead to internalized stigma about their HIV status, especially for those that have not accepted their status. Another participant shared a similar sentiment, “*Someone who would have difficulties*, *I think is someone who has not accepted their status, and I imagine that they would have to wear that reader on their neck. So, I think that’s the person who would have difficulty.”* (postpartum, age 31). Several participants expressed that, for individuals living in a setting with heightened HIV-stigma, feelings of shame may prevent use of the DPS because the Reader is markedly visible. For example:

*“I think the reader is fine, but since it will be visible when you are wearing it, I think other people would be ashamed, or would not be comfortable with wearing it, but I would not have that problem. I’m not ashamed, but I think other people would be ashamed.”* (postpartum, age 31).

Another woman suggested that people may ask what is around their neck, referring to the Reader, which could then lead to shame, “*It’s just that people are going to be asking me like, what do you have on your neck?…Maybe they will be ashamed to have this thing around their neck and people will be looking at them.”* (pregnant, age 32). The potential stigma that may arise from the DPS could then lead to reduced adherence and higher rates of MTCT, defeating the purpose of the DPS. One participant discussed a potential situation that could lead to non-adherence,

*“Since some people are hiding their status, it would be difficult for them to wear that reader while they are with other people and take their medication, so the person would want to have their own private space before they can take the dose. So, a person would not want to put that reader on their neck while people can see it.”* (pregnant, age 36).

Although the DPS was well-received by the majority of women in this sample, some discussed that wearing the Reader and needing to take their ARTs in front of others could delay pill-taking, as they would prefer to wait until they are in a private space.

## Discussion

This qualitative analysis explored preliminary acceptability and feasibility of the DPS technology to monitor ART adherence during pregnancy and postpartum among women in SA. Participants generally viewed the DPS favorably, noting that the DPS would improve their ART adherence and increase their accountability to prevent HIV transmission to their infants. The DPS was also viewed as a tool that could enhance motivation and communication with healthcare providers. However, the visibility of the Reader and its potential to disclose HIV status was a primary concern among this population. This investigation therefore supports future pilot work of the DPS in SA among pregnant and postpartum women. It also suggests key design features for the Reader that should be considered for implementation of DPS as an adherence measurement in this context.

Participants described that the DPS could reinforce adherence behaviors during the peripartum period, with many linking the primary motivation to adherence with their desire to protect their infant from acquiring HIV. Prior research has also demonstrated that having tangible, real-time data from the DPS could improve accountability and is well-suited to form long-term adherence behaviors (29). Tracking ART intake allows individuals to become aware of their own adherence patterns, leading to strengthening routine adherence, while also improving adherence when in nonroutine circumstances (i.e., staying at a friend’s house) (30). Among TB patients in Uganda, for example, participants found that digital adherence technology improved their medication adherence; monitoring their adherence allowed them to demonstrate their commitment to their regimen (31). Similarly, in our study, participants expressed motivation to share their adherence data with their healthcare team, not only to validate their consistency, but to open communication for productive feedback. Additionally, in the absence of a strong social support system, participants viewed the DPS as an alternative form of instrumental support to remind them to collect and take their ART. In other studies among PWH, being able to demonstrate commitment to treatment provided a strong sense of meaning, which DPS may too be able to foster (32, 33).

Real-time monitoring may be especially fitting to support ART adherence in the postpartum period, where adherence tends to decline due to newborn fatigue, structural barriers, and stigma. Fear of MTCT of HIV is a known motivator for ART adherence, yet pregnant and postpartum women often have reduced adherence to ART, especially in the postpartum period (34). In this analysis, many participants noted that the DPS would mitigate their fears of transmitting HIV to their baby because they could track their adherence in real-time. Utilizing DPS can help pregnant and postpartum women reduce their worries about forgetting to take their ART. In this context, future versions of the app could help reduce the barriers to adherence by displaying all future clinic appointments, for themselves and their baby, and providing consistent reminders to take their ART. As in previous digital adherence interventions (35), pregnant and postpartum women in the current study were also interested in a linked phone app because it would add a layer of support to collect and take their ART, as well as attend clinic appointments.

Despite high overall acceptability, participants identified stigma and concerns about inadvertent HIV status disclosure as potential barriers to consistent DPS use. Although most of the women in this sample had disclosed their HIV status to close friends and family, participants articulated that women who have not disclosed their status may have a difficult time wearing the Reader, as it may lead to uncomfortable questions or discrimination from others, evoking fears of stigma and discrimination. While only briefly discussed in interviews, participants who owned a smartphone or shared one with a family member were generally comfortable with family members accessing their adherence data, but some expressed greater hesitation about friends having access due to fears of judgement. Additionally, in SA, concerns about being seen at the clinic are a significant barrier to accessing HIV services and can be exacerbated by public use of medication or ART-related devices (36). Similar to other studies that examined feasibility and acceptability of the DPS, participants were interested in a Reader that could be smaller, more discreet, and easier to integrate into a daily routine (37). These findings suggest that future iterations of the DPS may leverage smaller wearable technologies such as wrist-borne devices, which are currently being developed in the US (38). It also reinforces the importance of choice and description of the intended use of DPS to potential users and emphasizes the need to ensure data privacy both in masking medications (e.g., using the gelatin capsule as a strategy to mask the pill being taken) and anonymity/security within the smartphone app (e.g., enabling password protection for access, or deidentifying data to prevent disclosure of ART use).

To support implementation among pregnant and postpartum women, DPS will likely need to effectively serve as both an adherence tool and a platform for patient-provider communication. Tailored messaging, discreet device design, and customizable app features could help reduce disclosure risks, decrease MTCT rates, and improve health outcomes for both mother and child. Provider engagement is also critical here. Participants wanted their healthcare providers to view their adherence data to increase transparency, trust, and ART adherence, ultimately improving the provider-patient relationship, which has been demonstrated to be poor in previous SA-based studies (39, 40). Utilizing DPS during a high-risk period is an opportunity for providers to help patients improve their adherence, especially among patients who are not virally suppressed or report low adherence. Other studies on the DPS have yielded mixed results from a provider perspective, one in which providers indicated that DPS could lead to potential privacy invasions, increased workload integrating DPS into clinical care, or disruptions to the patient-provider relationship due relying on data and not self-report (41). In another study, providers expressed concern that using the DPS may hinder shared decision-making by solely focusing on the data and not patients’ experiences (43). Despite these findings, investigations in other countries have demonstrated that providers may be able to leverage data from DPS to tailor medication adjustments and counseling decisions surrounding adherence (42). Perspectives from SA-based antenatal providers should be explored in future work. As well, DPS implementation is likely costly for widespread rollout, suggesting pregnant and postpartum women who are virally unsuppressed and/or have adherence challenges should be the primary target population. However, future research is needed to further explore logistical and financial barriers to DPS implementation in SA. There are also several additional directions for future research based on the current findings, including pilot testing the DPS among pregnant and postpartum women to assess real-world feasibility and impact on adherence and viral suppression, integrating patient and provider perspectives to tailor the DPS for this context, and redesigning DPS technology to increase acceptability in high-stigma settings.

This study has several limitations. First, we explored attitudes toward hypothetical use of the DPS, not experiences of actual use. Although this provides preliminary insight into the desirability of the integration of DPS into HIV care among pregnant and postpartum women, we cannot draw concrete conclusions about the true acceptability and feasibility of the technology from these interviews. Additionally, the sample may reflect selection bias, as HIV is a highly stigmatized condition in this context, and those that have disclosed their status to others or have personally accepted their status may have been more willing to participate in this study compared to individuals who do not want to engage in conversation about living with HIV. Finally, some cultural nuance may have been lost in translation, as most of these interviews were conducted in isiXhosa and transcribed and translated to English. We worked closely with the SA-based team to ensure accurate interpretation, but there is a possibility some cultural framing was lost.

Despite these limitations, this study adds to the growing body of literature supporting the continued exploration of digital adherence technologies, especially for those that are virally unsuppressed or have low ART adherence during the peripartum period. The DPS paired with a smartphone app and provider access, could enhance adherence through increased motivation, reminders, and accountability—especially during high-risk periods marked by fatigue, forgetfulness, or limited social support. However, concerns around device visibility and fear of unintended HIV disclosure highlights the need for stigma-informed implementation, such as more discreet device designs. Ultimately, DPS is a promising tool to support ART adherence and prevent MTCT, but future research should focus on piloting in real-world settings and improving device design to improve usability and acceptability among pregnant and postpartum women.

## Data Availability

The raw data supporting the conclusions of this article will be made available by the authors, without undue reservation.
